# The Heterogeneity of Empathy: Possible Treatment for Anhedonia?

**DOI:** 10.3389/fpsyt.2019.00185

**Published:** 2019-04-09

**Authors:** Sharee N. Light

**Affiliations:** Positive Affective Neuroscience Laboratory, Department of Psychology, Georgia State University, Atlanta, GA, United States

**Keywords:** empathy, empathic concern, positive-valence empathy, prefrontal cortex, ventral striatum, reward

## Abstract

Traditionally, empathy has been described as a process by which an individual “tries on” the negative emotion of others (i. e., empathic concern). A corpus of empirical work has been devoted to the study of this particular form of empathy. However, in this paper, the *heterogeneity model of empathy* is proposed as a method for counteracting the lack of attention paid to “positive-valence empathy”—our ability to respond to the negative and positive emotion of others with appropriate positive affect. Both empathic concern and positive-valence empathy are argued to have distinguishable behavioral manifestations and at least partially distinguishable neurobiological underpinnings. The potential value of positive-valence empathy induction for therapeutic purposes is also discussed.

Various researchers have defined the term empathy differently because psychologists and neuroscientists alike have had trouble coming to a consensus about the scientific meaning of the term. As a result, it has been difficult to formulate, and test theories of empathy in a rigorous fashion. The lack of a clear definition of empathy has made it particularly difficult to pair the behavioral manifestations of empathy with their neurophysiological correlates. Working from a phenomenological definition of empathy would be useful for generating specific, testable hypotheses—enabling researchers the opportunity to systematically determine how well each one aligns with known brain circuitry. This *Perspective* piece has the following structure:

- The concept of empathy as it is traditionally studied is presented.
- An alternative definition of empathy is presented that is more comprehensive (i.e., inclusive of positive-valence empathy).
- Predictions/premises of this reformulated definition/theory of empathy are proposed.
- Neurobiological evidence supporting the proposed reformulation/conceptualization of empathy is presented.
- Discussion of the treatment potential, or usefulness of empathy inductions for therapeutic change, is presented.
- Remaining empirical questions are presented.

## The “Standard” Empathy Definition vs. A Non-canonical Approach to Empathy

Most often, the term *empathy* is used to refer to the vicarious sharing of another's pain or sorrow (1). This form of empathy has been described as *empathic concern* (2–4) because the empathizer “shares in” the negative emotional experience of the target. However, the main argument made here is that positive-valence forms of empathy exist and should be studied alongside empathic concern because they may be useful in clinical settings as a standalone or adjunctive tool to well-established forms of behavioral treatment (e.g., Cognitive Behavioral Therapy-CBT) for positive affect deficits that are commonly observed in individuals with various psychological (e.g., Major Depressive Disorder-MDD, schizophrenia) and/or neurological (e.g., Parkinson's disease) disorders.

There are at least two forms of positive-valence empathy. An individual may exude positive emotion while in the presence of someone who is experiencing a negative emotional state as a means to convey tenderness and comprehension of the person's physical or emotional pain or sadness; in order to catalyze a positive emotional state in the target (e.g., an observer says “cheer up” to someone who has done poorly on a test). In contrast, an individual may exude positive emotion as a means to induce a state of joy in another person who is in a neutral emotional state, for its own sake. These are examples of *empathic cheerfulness*. Similarly, an individual may vicariously experience pleasure in response to someone else's positive emotion (e.g., the observer “shares in” another's joy). This is *empathic happiness*. Collectively, *empathic happiness*, and *empathic cheerfulness* can be referred to as “positive-valence empathy” (5, 6). In general, the exchange of happiness may ultimately serve to amplify—or up-regulate—the experience of happiness for one or both of the individuals involved, increasing *hedonic impact* (7). In fact, during *positive-valence empathy*, the observer and the target stand to generate “new” and heightened positive emotion that they may not otherwise have experienced if it were not for their interaction.

These concepts stem from the ancient Buddhist ideas of “sympathetic joy” and “loving-kindness” (8). In this tradition, positive-valence empathy can be acquired through intense mental training via meditative practice. The concept of *positive-valence empathy* was also later discussed by historical figures such as Theodor Lipps (9) and Adam Smith. For example, in 1759 Adam Smith wrote in *The Theory of Moral Sentiments*:

When we have read a book or poem so often that we can no longer find any amusement in reading it by ourselves, we can still take pleasure in reading it to a companion. To him it has all the graces of novelty; we enter into the surprise and admiration which it naturally excites in him, but which it is no longer capable of exciting in us; we consider all the ideas which it presents rather in the light in which they appear to him, than in that in which they appear to ourselves, and we are amused by sympathy with his amusement which thus enlivens our own [excerpt taken from Goldman (10)].

Though Smith uses the word “sympathy” in this passage, an argument could be made that the description illustrates our present day definition of *positive-valence empathy*. Importantly, here empathic cheerfulness is conceptualized as being separate from prosocial behavior; at least based on the results of prior research. Namely, in previously unpublished aspects of the [Light et al. (6)] data set, the correlation between *trait* empathic cheerfulness (as measured by an independently validated self-report scale) and prosocial behavior (operationalized as selection of actual children's books for donation) was non-significant (*r* = −0.009, *p* = 0.94); and similarly, *task* empathic cheerfulness (measured via self-report as participants viewed a television program that tends to elicit empathy) did not predict subsequent prosocial behavior (*r* = −0.036, *p* = 0.769). This provides preliminary evidence in a healthy adult sample that empathic cheerfulness is distinct from prosocial behavior. Though *trait* empathic cheerfulness does correlate with *trait* empathic concern (as measured by the Interpersonal Reactivity Index-IRI) (*r* = 0.574), and *task* empathic cheerfulness correlates with *task* empathic concern (*r* = 0.611) (6).

Given the existence of positive-valence empathy, a reformulated model of empathy seems necessary [e.g., (11)]. The “standard” model of empathy, which generally equates empathy with affective sharing, is lacking primarily because it conflates emotion identification (i.e., the ability to correctly “read” or interpret the emotion of another) with affect sharing (the ability to feel *with* someone else) [see (11)]; and the entire model is generally based on the singular observation of empathy for *physical* pain. Our working model of empathy needs to evolve to reflect our expanding understanding of the above mentioned empathy subtypes and their underlying neurobiology. In other words, although the neurocircuitry that forms the basis for our ability to empathize with the *physical* pain of others has been well-elucidated to date, it alone fails to explain more recently evolved subtypes of empathy.

Our empathy ability for additional, more complex emotional states than physical pain likely evolved *after* the mechanism responsible for our ability to empathize with the physical pain of others (i.e., from an evolutionary perspective). Furthermore, the underlying neural mechanisms involved in these more complex, later evolving empathy subtypes likely relate to the functioning of neural circuitry that layered over—or emerged from—the basic building blocks of the pain circuit (12, 13). Thus, there is an evolutionary-based and neural-based cause for continuing to expand our conceptualization of empathy.

Just as basic principles of affective neuroscience (and even cognitive neuroscience) were initially formulated based on attributing psychological functions (e.g., “emotion,” “memory,” “language”) to specific brain regions, these fields have evolved over time and now take much more of a systems approach to understanding psychological constructs. Similarly, given that the corpus of empathy research has focused on one subtype of empathy for a long time (i.e., empathy for physical pain), the resulting theories of empathy have generally been limited by this conceptual focus. Here, an attempt is made to promote an empathy model that is more comprehensive and can account for all known subtypes of empathy; with the idea that common and dissociable neural circuitry evolved from the pain circuit are involved in all empathy subtypes.

Therefore, the primary question is how best to (parsimoniously) account for/explain both empathic concern on the one hand, and positive empathy subtypes on the other hand. The fundamental threads of such an inclusive and refined model have already been articulated [e.g., (11, 12)]. Specifically, Coll et al. (11) suggest that empathy should refer to the degree to which the empathizer's emotional state matches that identified in the target, though this emotional response may deviate from the target's *actual* emotional state. Using this definition, empathy can be measured as a single process, rather than as affective identification + affect sharing. However, here it is argued—given the existence of empathic happiness and empathic cheerfulness—even this Coll et al. (11) model stops short. Specifically, we know that empathic cheerfulness occurs on a time-scale consistent with empathic concern (as does empathic happiness), but uniquely does not predict prosocial behavior (as noted above) in the same way that empathic concern or empathic happiness does. Given these facts, it seems likely that empathic cheerfulness does represent a “true” alternative response to the physical or emotional pain of someone else (e.g., other than empathic concern or personal distress), and thus should be included in any explanatory model of empathy. That said, the definition of empathy should make reference to the fact that the empathic response is an emotional reaction that is both relevant and other-oriented in relation to the emotional state of the target (and can be either positive or negative in valence). For example, I may perceive someone who has lost an adored pet, and thus infer that they are likely feeling sad. At this stage of the process, I may either show one of three potential empathy–related responses: (a) personal distress, (b) empathic concern, or (c) empathic cheerfulness. Important to note here, the experience of empathic cheerfulness can occur without a corresponding prosocial act (i.e., the experience of empathic cheerfulness need not involve an overt helpful act). For example, in the situation described above, where the empathizer encounters someone who they perceive to be feeling sad, the empathizer may smile and say “cheer up” without there being a subsequent overt helpful act (e.g., a pat on the back). Overall, the model proposed here deviates from Coll et al. (11) in that it is not required that the emotion “expressed” or consciously “felt” by the empathizer—on a time scale of seconds to minutes—has to even be particularly similar (e.g., in valence, intensity, or quality) to that which is perceived to be present in the target. The emotion need only be *other-oriented, relevant*, and clearly *reflective*/*resultant* from the empathizer's own apprehension/*comprehension/interpretation* of the target's emotional state.

Relatedly, in naturalistic settings, such as the psychotherapy room, researchers have already found that defining empathy narrowly as affective matching is not particularly explanatory or helpful. Specifically, Elliot et al. (14) found that when it comes to measuring the flow of empathy in a therapeutic relationship (i.e., between a client and their psychotherapist), empathic accuracy (i.e., my affect matches yours) does not significantly account for variance in treatment outcome. This provides further support for the idea that conceptualizing empathy as literally “accurate emotional state matching” is limiting, and we should move away from definitions of empathy that advocate this conceptualization.

The Coll et al. (11) model also stipulates that empathy has occurred only when the empathizer *consciously* identifies the emotional state of the target and has an affective sharing experience; whereas personal distress results when the emotion of the target is *unconsciously* identified and affect sharing ensues. The proposed model does not include this conscious/unconscious distinction because, again, empathic cheerfulness appears to be a valid empathic response, but it violates this tenet (at least in certain instances). For example, someone may unconsciously perceive the emotional state of a target who has lost their pet (e.g., sadness), but they actually may only (or predominantly) experience and exude positive emotion, as in empathic cheerfulness. Data pertaining to the minute timescale of emotional responsiveness, and the miniscule timescale with which the underlying neurobiology of emotion can unfold, supports this assertion (15–17).

### Re-conceptualizing Empathy: The Heterogeneity Model of Empathy

Under the umbrella of the “*heterogeneity model of empathy*,” the following general definition of empathy is offered**: empathy refers to a** ***change*** **in emotional state triggered by the formation of an internally generated replica of the emotional state of another**. The integration of information held in mind about the observer's own emotional state with information held in mind about the emotional state of the target provides the substrate for the onset of an entirely new emotional state in the observer. **In addition to the creation of this new vicarious-based emotional state, an other-oriented feeling of goodwill may also be generated in the observer, and this may include (or rise to the level of) feelings of enjoyment in certain instances**. Together, this package is empathy (18). There are several assumptions embedded in this definition.

First, for empathy to occur, the observer must hold at least two mental representations in mind simultaneously: one that contains information about their own emotional state and one that contains information about the emotional state of the target. Furthermore, this information needs to be kept in an online, highly accessible state for a brief period of time *while* the information is also being actively manipulated. Therefore, at certain times the observer must hold a mental image of what the target is feeling on the one hand, and hold a mental image of their own feelings on the other hand, while the situation is actively unfolding (19). This can be unconscious.

Second, it is proposed that the replica of the emotional state of the target that takes shape within the observer is built using several forms of information, including: (a) conscious or unconscious information taken in through the senses about the target's emotional state (e.g., facial expression, vocal tone, body posture), which is based on simulation theory (20), and (b) “as-if” information (e.g., this is self-generated information that the observer obtains by imaginatively placing him or herself in the target's shoes and “trying on” their emotional state; for example the observer may ask themselves “what would it actually feel like if x occurred?”), and (c) “if-then” information (e.g., applying tacit rules about the cause-and-effect relationship between events and emotional states; for example, a tacit rule may be “generally people feel happy [emotion] when they receive a gift [event].” This is consistent with the Theory of mental state attribution (21). For example, if I hear that my friend received a gift from her mother, I can apply this general rule and infer that my friend likely feels happy.

Third, similar to Coll et al.'s (11) and Stotland's (22) definition of empathy as “an observer's reacting emotionally because he perceives that another is experiencing or is about to experience an emotion” [pp. 272; (22)], this conceptualization of empathy does not assume that the observer's emotional experience will be an *exact* replica of the target's emotional state. In fact, the definition only asserts that the observer's emotional state *changes* from its original state as a result of the impact caused by internally representing the emotional state of someone else. In other words, the dual representation of emotional states (i.e., the observer's own and that of the target) can cause the observer's mental/emotional state to *change*.

Finally, this definition does not restrict the use of the term *empathy* to situations in which the observer shares in the negative emotion of another. Instead, the definition allows for the term *empathy* to be applied when an observer shares in the positive emotion experienced by a target. In fact, this definition allows for the term *empathy* to be applied even if the observer experiences an emotion that differs in valence from the emotional state of the target. Stotland (22) referred to this special case of empathy as *contrast empathy*.

### Predictions of the Heterogeneity of Empathy Model

In general, the so-called “empathy circuit,” which tends to include the anterior cingulate cortex and anterior insula most prominently, is often described in the literature regarding the experience of empathy for *physical* pain [e.g., (23)]. Although prefrontal cortex activation is also generally reported in these studies, it is not emphasized [e.g., (23–25)]. **The heterogeneity model asserts that although this prefrontal cortex (PFC) activation is often overlooked, it likely plays a more prominent role in empathy processes than currently thought, particularly in empathy for emotional states that do not involve physical pain (e.g., emotional pain, happiness, etc.)**. This is hypothesized because, in general, the PFC organizes information from lower levels of processing (e.g., the limbic system, sensory systems) and uses that information to orchestrate thought, emotion, and motor actions in accordance with internal goals (26). Specifically, the prefrontal cortex plays a central role in both emotional processing and executive functioning, making this region particularly interesting to study in relation to empathy because the occurrence of empathy—as described above—likely increasingly depends upon (across the lifespan) the ability to hold emotional information in mind (i.e., a working memory function) and orchestrate, step-by-step, an appropriate emotional response.

Given the hypothesis about the role of dual representation of emotional states in the empathizer, a significant role of the dorsolateral prefrontal cortex (DLPFC), extending into the frontopolar prefrontal region, is suspected to occur in all forms of empathy (particularly those that do not involve physical pain), to some degree. A review of recent anatomical, neuroimaging, electrophysiological and developmental findings (presented below) support the existence of a rostro-caudal hierarchy in the prefrontal cortex, with the frontopolar cortex processing more abstract information than the dorsolateral region, and the two regions being heavily interconnected (27). Both regions show activation during empathy tasks. The dorsolateral prefrontal cortex may become activated when representing multiple emotional states, whereas the frontopolar region may be recruited to oversee the completion of higher order goals relevant to empathic interpretation and execution of empathic behavior. The following evidence exists to support the idea that the dorsolateral and frontopolar prefrontal cortex may collaboratively contribute to such empathic ability; across both empathic concern and positive empathy.

*Evidence Supporting A Role for the PFC in Empathic Concern*. Singer et al. (23) found that adult participants showed significant activation in the dorsolateral prefrontal cortex (BA 47) when viewing their romantic partner receive a painful stimulus. Interestingly, this pattern of prefrontal activity was not present when these participants received the painful stimulus themselves. In fact, participants did not show any significant prefrontal activity when they were the direct recipients of a painful stimulus. This result implicates the dorsolateral region of the prefrontal cortex in empathic processing. The dorsolateral activity observed in this study may be an indication that the observer registered the emotion of the other person. This view is supported by other data that suggests that the dorsolateral PFC region is involved in holding internal representations of external stimuli. The ability to form and hold an internal representation of someone else's emotional state may provide a means for the observer to experience some kernel of that same emotion (28, 29).

Activity in the frontopolar cortex has also been found to relate to negatively-valenced empathic emotion. For example, using a neuroimaging paradigm, Jackson et al. (30) found that there was a significant increase in frontopolar activity (BA 10) when adult participants thought about someone else's pain. Furthermore, Ruby and Decety (31) found that the frontopolar cortex became more active when adult participants had to respond to emotionally evocative situations from the perspective of another person compared to when participants had to take a first person perspective. Additionally, using a cross sectional design, Decety and Michalska (32) found that adults demonstrated greater dorsolateral and ventrolateral activity during an empathy induction task relative to children and adolescents, suggesting that adults rely more heavily on attention and cognitive control circuitry in empathic situations (33).

Similarly, across 4- to 8-year-olds, affective empathy (the ability to be emotionally reactive to the emotional displays of others) related positively with dorsolateral activation in older children relative to younger children (34). This set of findings suggests an increase in recruitment of dorsolateral prefrontal cortex with advancing empathy ability (34). Similarly, in a study with individuals with traumatic brain injury, damage to the dorsolateral prefrontal cortex was found to significantly diminish one's ability to perceive (via the human face) emotion and use emotional information to make interpretations (35).

*Evidence Supporting A Role for the PFC in Positive-Valence Empathy*. In a study of the neural correlates of charitable donation, fMRI was used to visualize brain activity while people played computer games by which they could earn money for real-life charities. The results indicate that the “joy of giving” has an anatomical basis in the brain—the same one that exists for other types of reward (e.g., food, sex, money)—and can be found in the ventral striatum (36). However, importantly, activity in the frontopolar cortex related participant's report of their feelings of joy and everyday charitable involvement (36).

Electrophysiological and neuroimaging data also suggest that increased activity in the dorsolateral [(37–40), orbitofrontal (41), ventrolateral (42), and frontopolar prefrontal cortex (43) relate to the subjective experience of basic positive emotions such as happiness and/or pleasure, which is linked to increased empathy behavior (44). Results from an electroencephalographic (EEG) study involving children aged 6–10 years old (5) suggest that children who tend to exhibit *empathic concern* vs. *positive empathy* have distinct neurophysiological profiles during the elicitation of pleasure (on a completely separate day). For example, children who demonstrated substantial behavioral *empathic happiness* exhibited relatively symmetrical co-activation of dorsolateral and frontopolar prefrontal activity via EEG when they were exposed to a positive stimulus on a separate day (5). The sustained maintenance of equal amounts of left and right lateral and anterior prefrontal cortex activity over the course of a positive stimulus may indicate that these children bring both lateral frontal hemispheres to bear on pleasure-inducing tasks. Children who demonstrated a high level of behavioral *empathic concern* exhibited right-sided *and* then left-sided prefrontal activity across the lateral and anterior-most regions of the prefrontal cortex during a positive affect inducing task (5). The ability to exhibit right and then left-sided prefrontal activation during a positive task might relate to an ability to flexibly experience (or shift between) positive and negative emotional states during a task, and may be facilitated by amygdala connectivity (15). If a child can flexibly experience negative and positive emotional states, this general ability may enhance their ability to internally represent the emotional states of others and respond to the negative emotion of others with a combination of negative emotion (e.g., sadness and concern) and/or positive emotion (e.g., positive empathy, goodwill). The results from a separate behavioral study involving children under the age of 2-years-old provide evidence that young children experience greater happiness when giving treats to others rather than receiving treats themselves (45), further supporting the role of positive emotion in empathy.

Finally, a neuroimaging study of vicarious reward revealed that adult participants who reported enjoying observing other people winning a game show exhibited greater bilateral frontal pole activation, in addition to ventral striatum activation (46). Ventral striatum activity also increased when the participants played the game themselves and won, but prefrontal activation was absent. Again, these results support the findings of Light et al. (5) because a dorsolateral-frontopolar prefrontal circuit was implicated in empathy; in this case, positive-valence empathy.

*Empathy Circuits*. It is important to note that the dorsolateral and anterior-most regions of the prefrontal cortex do not work in isolation to enact empathic feelings or behavior in adults (or children), and these different regions may be distinguishable in terms of what they *do* in the various empathy subtypes. For example, it is hypothesized that the dorsolateral prefrontal cortex facilitates the dual representation of the emotional state of the observer and the target. However, as the emotions that need to be represented become more abstract, it is likely that the frontopolar cortex becomes more active (36).

**Figure 1:**
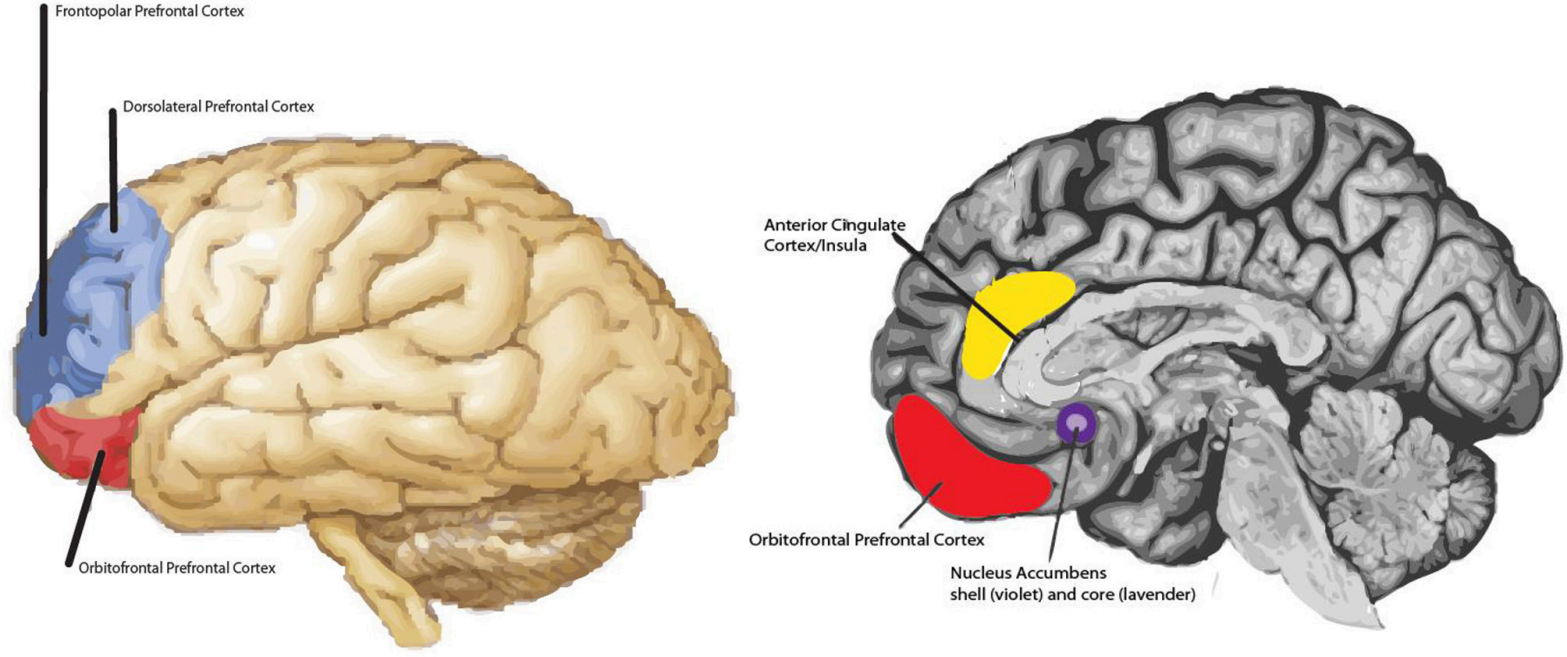
Main regions hypothesized to be involved in non-physical pain empathy processes. For example, frontopolar prefrontal cortex is shaded in dark blue and the dorsolateral prefrontal cortex is shaded in light blue. Both regions may be involved in empathic concern and positive-valence empathy. The extent of prefrontal activation and lateralization effects may distinguish one form of empathy from another; in addition to differential patterns of subcortical activity (e.g., nucleus accumbens vs. amygdala). Note: Amygdala not highlighted.

Both prefrontal regions are likely active in empathic concern and both forms of positive empathy. What may distinguish the different subtypes of empathy are the extent of prefrontal activation, different patterns of lateralization of prefrontal activity [see (5, 40)], and/or differential patterns of concomitant subcortical activation. Altogether, several primary brain structures are hypothesized to be involved in the various empathy subtypes, including: the amygdalae, the ventral striatum (e.g., nucleus accumbens and globus pallidus), anterior cingulate, insula, and the dorsolateral and frontopolar prefrontal cortex (see Figure 1). Bottom-up and top-down processing among these structures may contribute to our capacity to resonate with each other emotionally and experience the various empathy subtypes. Bottom-up processing allows for the rapid processing of an affective signal, such as someone in pain (39; 26); and top-down processing allows for the perceiver's intentions, motivations, and feelings to be attached to the comprehended feeling state initiated by the bottom-up process. In adults, signals sent from the amygdala and/or anterior cingulate/insula to the lateral and anterior-most prefrontal cortex are thought to form the basis of the bottom-up empathic processes, while activity originating in prefrontal regions is thought to play an important role in top-down empathic processing (47). There is likely a bi-directional route by which the lateral aspect of the prefrontal cortex not only is involved in processing the initial affective signal generated by the amygdala (i.e., which carries information about the emotional state of the target) as proposed by Decety (47), but also plays a role in generating the first kernels of the other-oriented feeling of goodwill in the empathizer, a form of positive affect, that may get elaborated by prefrontal cortex. It is hypothesized that the prefrontal cortex is largely responsible for the higher order feeling of goodwill that is central to the proposed model of empathy, though subcortical structures likely contribute too, such as the nucleus accumbens, which is involved in reward.

### Treatment Potential

The proposed “heterogeneity of empathy” model is based on preliminary work that indicates that *increased* empathy (all types) predicts *increased* positive emotionality (5); and positive affect is associated with many desirable outcomes, such as problem solving, well-being, longevity, and reduced likelihood of dementia. Thus, understanding the idiosyncrasies between various empathy *subtypes* and the symptom of anhedonia (i.e., the reduced ability to experience pleasure) could perhaps inform new therapeutic approaches that make use of empathy induction paradigms as a means to reduce anhedonia; especially given the ethics of increasing positive affect—i.e., the need to increase positive affect without use of potentially addictive medication or unhealthy increases in risky behavior.

Although anhedonia is an elusive construct to study, most agree it is a symptom that everyone would like to decrease/eliminate. In its place, most would rather experience “happiness.” Happiness can be defined as the frequent experience of positive emotions (48–50) and is trait-like (but is not as stable across time as most would assume, with ~33% of variance in happiness being accounted for by an unstable state/error variable) (51). Its neurobiological substrate is rooted in the functioning of frontostriatal circuitry; and subjective happiness has been linked to orbitofrontal and dorsolateral prefrontal cortex, and ventral striatum activity most commonly (52–55). It is still somewhat unclear whether happiness and anhedonia exist as opposite ends of a continuum or represent distinct constructs, each with their own continuum; however, longstanding research suggests that anhedonia is a viable candidate for an underlying endophenotype for psychiatric/neurological dysfunction, given that it appears across several psychiatric and neurological disorders, is trait-like itself, and also has neurobiological underpinnings focused in frontostriatal activation (56). In other words, it fits within the Research Domain Criteria (RDoC) framework put forth by the National Institute of Mental Health (NIMH) (57). “RDoC” is a research framework for new approaches to investigating mental disorders. It integrates many levels of information (from genomics and circuits to behavior and self-reports) in order to explore basic dimensions of functioning that span the full range of human behavior from normal to abnormal. One of the RDoC domains is “Positive Valence Systems,” and part of the urgency in studying this domain is due to the fact that there is a strong need for novel/alternative methods of *inducing* positive affect (e.g., in various clinical populations) given the difficulty of safely and repeatedly increasing positive affect without the use of medication or other substances, or without having people engage in unhealthy, risky behavior.

For example, although treatments such as Behavioral Activation (BA) are effective in treating MDD—and despite its focus on scheduling pleasant events—anhedonia remains a residual symptom in a large subset of these patients, particularly once the treatment is stopped (56, 58). Attempting to directly increase positive emotion has proven difficult in clinical populations. This is also reflected in the research literature, which demonstrates the difficulty of inducing a robust positive affective response in laboratory settings. Given these limitations, and our own work suggesting that some MDD patients may have a tendency to unconsciously suppress positive emotion (42) (at least in certain situations), systematically inducing empathic happiness offers an alternative route to help people decrease anhedonia.

Inducing empathic happiness may have treatment potential most likely as an adjunctive treatment to Cognitive Behavioral Therapy (CBT) or Behavioral Activation (BA). Behavioral Activation (BA) is a treatment that has been shown to be effective in treating Major Depressive Episodes (59), and its mechanism of action is primarily thought to relate to the scheduling of pleasant events. This treatment is primarily based on addressing *anticipatory anhedonia*, or the reduced ability to experience pleasure in the pursuit of pleasurable activities. However, the treatment generally does not directly address *consummatory anhedonia* (i.e., the ability to enjoy rewards in-the-moment once obtained). Indeed, anhedonia remains a residual symptom at a rate that is similar to other behavioral treatments that do not necessarily focus of positive affect at all. Therefore, attempting to directly increase/train the subjective experience of, and sustenance of, positive emotion via positive-valence empathy induction specifically in response to the *attainment of a reward* (i.e., consummatory pleasure) may be a useful adjunct to lower the rate of persisting anhedonia in this clinical population.

Through the induction of empathic happiness (and possibly empathic cheerfulness), “new” positive emotion may be produced in the empathizer; and given the route of induction, it may be more sustainable than any positive emotion invoked by medication use or engagement in currently available behavioral remedies. This is primarily because the positive emotion produced through empathy induction will likely both quantitatively (a) increase subjective positive emotion (i.e., increase *hedonic impact*) and (b) increase the execution of prosocial behaviors in the community at large (increasing social connectedness more broadly). This is predicted because the underlying biological mechanism/route to increased positive emotion is likely different; with empathic happiness induction being more focused on the experience of pleasure vs. just increasing the *number of* pleasant activities. Teaching individuals to enjoy *obtained* rewards (i.e., consummatory pleasure) in a new way is in contrast to Behavioral Activation's focus on the *pursuit/scheduling* of pleasurable activities (i.e., anticipatory pleasure). The proposed empathy induction process likely maps onto a separable fronto-striatal circuitry (possibly mediated by broader prefrontal involvement) and neurotransmitter system functioning than BA alone; given animal models showing that consummatory and anticipatory positive affect are mediated by separable neurotransmitter systems (i.e., dopamine vs. opioids) and neural circuits. Opioids relate to consummatory pleasure, which is likely emergent from its role in the pain analgesia system.

Thus, the proposal here is that a brief intervention designed to teach individuals to engage in empathic happiness represents a novel technique for treating anhedonia in-the-moment, and is based on a literature that suggests that the hedonic treadmill hypothesis is not entirely accurate as originally conceptualized (54) in that the generation of prosocially-mediated positive affect may be quantitatively greater (i.e., increased hedonic impact) and more resistant to tolerance effects across time because it may tap a slightly different, broader fronto-striatal circuitry than self-focused “discrete” joy or anticipatory pleasure does. The proposed intervention relies on the popular and well replicated finding in the literature that most people feel quantitatively *more* happiness when they engage in other-oriented, prosocial activities (60) opposed to self-focused activities. Capitalizing on this phenomenon, tailor-designed empathy inductions could be carried out with patients to maximize hedonic impact. Learning to effortfully evoke/increase empathic happiness using behavioral techniques such as “savoring” (i.e., relishing in one's positive experiences) and “capitalization” (telling someone/sharing the good things that happen in life) may serve to ultimately diminish anhedonia (60)—via novel activation of previously dysregulated neurotransmitter systems and dysregulated nodes of fronto-striatal circuitry—and promote longer-term/sustainable happiness.

The hypothesis that empathic happiness may be harnessed as a treatment—or adjunct to established treatments such as Behavioral Activation—fundamentally stems from the belief that empathy is mutable, and anhedonia may be altered via targeted modification of aspects of fronto-striatal activation by behavioral means, such as a successful empathy induction. The idea that empathy is mutable stems from a small but growing empirical base. Research findings suggest that interventions derived from ancient contemplative practices that focus on increasing traits such as compassion (e.g., through the practice of “loving-kindness meditation”)–a construct related to empathy–can induce plasticity-related alterations in the brain, and these alterations support a range of positive behavioral outcomes such as improved immune function, increased prosocial behavior, and enhanced problem solving ability (61). Thus, the experience of “empathic happiness”—under normal circumstances and by definition—should increase the experience of positive emotion in vulnerable individuals (i.e., particularly in anhedonic individuals). It is important to note that positive empathy is not expected to be a panacea of any sort. It is only expected that certain patients in particular, i.e., patients that suffer with anhedonia and other residual symptoms following “successful” treatment of MDD (either pharmacological and/or behavioral), may be more impacted by this type of training vs. individuals who demonstrate a different symptom pattern. However, this technique could be useful for several disorders characterized by anhedonia; not just MDD.

Empathic happiness and empathic cheerfulness may also be beneficial to professional providers of care. This idea stems from results from a recent neuroimaging study. Engen and Singer (62) found that individuals trained in compassion-based emotion regulation vs. cognitive reappraisal demonstrated increased positive affect (vs. dampened negative affect in the cognitive reappraisal condition) and this subjective experience was reflected by increased activity in ventral striatum and medial orbitofrontal prefrontal cortex in the compassion-based emotion regulation group. Compassion relates to empathy and can be defined as a feeling of concern for the suffering of others that is associated with the motivation to help (63). Essentially, these results suggest that the elicitation of compassion-based emotion regulation circumvented the experience of personal distress. This finding has implications for how to reduce caregiver burnout. However, it would also be interesting to determine whether training caregivers to engage in empathic happiness and empathic cheerfulness in relation to their clients—and then training clients to engage in empathic happiness in their everyday lives—may also be an effective and alternative route for reducing caregiver burnout, but may also directly facilitate therapeutic change in the client.

The primary techniques that clinicians (and clients) can be taught to use include basic theory-of-mind/emotion decoding, “emotion regulation/cognitive reappraisal” (64) and “capitalization/savoring” (60). For example, in order to practice “capitalization/savoring,” empathizers can go through training in which they learn how to better concentrate on the positive characteristics of others (14), including their clients. An example of how they can be trained to use the “theory of mind/emotion decoding” technique would look like this: “Focus on instances when you noticed an individual smiling. Try to notice whenever the individual smiles, and try to imagine how good the person may feel at those moments; and inquire about their emotional state at those moments.” As an example of an “emotion regulation/cognitive reappraisal”-based strategy, empathizers can be trained to: think about what past happy memories may mean for that person, with particular emphasis on how they can manifest similar experiences in their current and future life. Overall, training empathizers to focus on thinking about people of interest in the most positive light possible could be effective for inducing more empathic happiness. Another example of “savoring:” prompt trainees (i.e., empathizers) to incorporate how they are feeling in the moment in reaction to their current experience, later in their day, or a week from now. Empathizers undergoing this type of “training” could be given an opportunity to practice these techniques as they view selected video clips from movies or television shows that tend to evoke positive empathy. For example, our prior work (6) indicates that reality television can be a useful elicitor of positive empathy, and could be used in a training context to “teach” positive empathy skills.

### Concluding Remarks and Future Directions

Using the lens of the *heterogeneity of empathy model*, it is suggested that frontostriatal reward circuitry plays a special role in empathy processing. Extending from this, several general statements can be made about the nature of empathy based on the empirical data currently available. First, the successful execution of empathic processes (both *positive-valence empathy* and *empathic concern* processes) likely involve activity in the prefrontal cortex; the lateral and anterior-most regions of the PFC seem particularly relevant given their particularly strong roles in working memory, abstract thought, and positive affect (5, 40).

According to ancient Buddhist teachings, “sympathetic joy”—the earliest known reference to positive-valence empathy—can be achieved through meditative practice (8). Similarly, according to Lipps (9), empathy is the result of a contemplative state that can result in enjoyment/pleasure; and as Adam Smith eloquently stated in *Theory of Moral Sentiments*: “How selfish soever man may be supposed, there are evidently some principles in his nature, which interest him in the fortune of others, and render their happiness necessary to him, though he derives nothing from it except the pleasure of seeing it (pp. 585).” In sum, evidence abounds that human beings have a capacity to relate to the emotions of others, including the positive emotions of others. Importantly, this process may be rewarding, just as food, money, and artwork are (65). In fact, there is some evidence to suggest that, at least in children, *greater* happiness can be derived from cultivating happiness in others rather than experiencing personal happiness (45). This leads to the final premise of the heterogeneity model: empathy may indeed be a rewarding process, and likely contributes to eudaimonia (i.e., well-being). Well-being, or the “good life,” can be conceptualized as having at least two dimensions: hedonic (e.g., the experience of moment-to-moment pleasure) vs. eudaimonic (e.g., the experience of positive meaning and/or sustained, long-term positive affect). We are only beginning to tease these constructs apart; but basic neuroscience research in hedonic processing suggests that heightened “liking” (i.e., pleasure derived from the *attainment* of a reward) is mediated by a relatively small set of brain structures that includes the nucleus accumbens shell and orbitofrontal prefrontal cortex, whereas subjective well-being or eudiamonia likely involves the prefrontal cortex more widely (7). Therefore, the intersection of prefrontal cortex activity, subcortical activity, short and long-term positive affect, and empathy may be an important cornerstone of prosocial behavior and heightened well-being.

“Positive-valence empathy,” if it can be taught, a currently hotly debated question in the empathic concern literature, stands as a potentially relevant clinical tool (66). Anhedonia, the reduced ability to experience pleasure, is a common symptom across various disorders including Major Depressive Disorder (MDD) and Parkinson's disease. Thus, training people to increase their “positive-valence empathy” skills may be an efficacious treatment technique against anhedonia, in addition to more traditional techniques for increasing “personal” positive affect (e.g., Behavioral Activation).

The future of the field of positive affect and empathy research will benefit from mainstreaming the concept of “positive-valence empathy” alongside “empathic concern.” Any comprehensive theory of empathy needs to account for the existence of “positive-valence empathy.” Work to date provides good evidence for a role of fronto-subcortical reward circuitry in complex empathy. However, future work will need to address the potential neurobiological link between empathy and well-being, and focus on elucidating the unique neurobiological contributions of “empathic concern” vs. “positive-valence empathy” subtypes to differential prosocial behaviors.

## Author Contributions

The author confirms being the sole contributor of this work and has approved it for publication.

### Conflict of Interest Statement

The author declares that the research was conducted in the absence of any commercial or financial relationships that could be construed as a potential conflict of interest.
